# Adding value for clients during work disability assessments: A qualitative exploration from the perspective of medical examiners

**DOI:** 10.3233/WOR-230305

**Published:** 2024-10-07

**Authors:** Marije E. Hagendijk, Zhouwen Tan, Marijke Melles, Jan L. Hoving, Sylvia J. van der Burg-Vermeulen, Nina Zipfel

**Affiliations:** a Department of Public and Occupational Health, Amsterdam UMC Location University of Amsterdam, Coronel Institute of Occupational Health, Amsterdam Public Health Research Institute, Amsterdam, The Netherlands; b Faculty of Industrial Design Engineering, Delft University of Technology, Delft, The Netherlands

**Keywords:** Vocational guidance, return to work, sick leave, value-based health care, delivery of health care, qualitative research

## Abstract

**BACKGROUND:**

Value-based healthcare delivery focuses on optimizing care provided by measuring the healthcare outcomes which are most important to the clients relative to the total care costs. However, the understanding of what adds value for clients during work disability assessment is lacking.

**OBJECTIVE:**

To explore what medical examiners (MEs) perceive as valuable during the work disability assessment process, by exploring possible: 1) facilitators, 2) barriers and 3) opportunities to add value for the client during the work disability assessment.

**METHODS:**

For this explorative qualitative study, 7 semi-structured interviews were conducted with MEs in the Netherlands. Thematic coding was performed for all interviews.

**RESULTS:**

A large variety of facilitators (*n* = 22), barriers (*n* = 17) and opportunities (*n* = 11) were identified and inductively subdivided into four main themes: 1) coherent process, including all time related aspects, 2) interdisciplinary collaboration, including all aspects related to the collaboration between the ME and other professionals, 3) client-centred interaction, including all aspects related to the supportive interplay from the ME towards the client, and 4) information provision on all aspects during the work disability assessment process towards the client to ensure a valuable work disability assessment process.

**CONCLUSIONS:**

The overview of identified possible facilitators, barriers and opportunities to add value for clients from the perspective of the ME may stimulate improvement in the current work disability assessment practice and to better match the client needs.

## 1 Introduction

Value-based healthcare (VBHC) focuses on optimizing healthcare outcomes that matter most to clients relative to the total care costs [1, 2]. The delivery of VBHC has been found to improve client outcomes and reduce inefficiencies in the healthcare system [3–5]. Therefore, with increasing strengthening of the VBHC rationale, in many, mostly high-income, countries value-based approaches are implemented in the healthcare systems [6, 7].

To date, the implementation of VBHC mainly focuses on curative healthcare, but is almost non-existent in occupational healthcare. As a result, the creation of value-based occupational healthcare lags behind. Nonetheless, because of the increasing number of workers with chronic diseases, declines in mortality rates and increase in retirement age in most countries, there is an increasing demand for guidance and support from occupational health [8–11]. A more prominent focus on the delivery of value-based occupational healthcare may enhance its quality despite the rising demands [12].

An important task within occupational healthcare for workers on long-term sick leave (from now on called clients) is the assessment of the client’s functional limitations and work disability. During this work disability assessment, a medical examiner (ME) assesses the client’s (dis)ability for work according to social insurance criteria and reports on the client’s working capacity and prognosis for functional recovery [13]. However, in order to add value for the client during the work disability assessment process, currently it is unknown how and what the MEs themselves perceive as valuable and how they believe value for their clients can be improved during the work disability assessment.

The objective of this qualitative study was to explore what the ME perceives as valuable during the work disability assessment process, by exploring possible: 1) facilitators, 2) barriers and 3) opportunities to add value for the client during the work disability assessment.

## 2 Methods

### 2.1 Design and setting

This qualitative explorative study was conducted as part of a larger research study investigating the possibilities of using the concept of VBHC in occupational healthcare. The study was conducted by researchers of Amsterdam University Medical Centres, who were responsible for the design of the research question, data analysis and development of this manuscript, in collaboration with Delft University of Technology, which provided students of Master Design for Interaction who conducted the interviews and co-analysed the data. The study was conducted and reported in accordance with the Consolidated Criteria for Reporting Qualitative Research (COREQ) checklist [14].

#### 2.1.1 Work disability assessment in the Dutch context

In the Netherlands, the ME conducting work disability assessments is the insurance physician, mainly working for the Dutch Social Security Agency (SSA). To establish the eligibility for a disability claim, an assessment by the insurance physician targets to determine disease-related functional limitations and assess (partial) work ability of the client according to pre-defined social insurance criteria [15, 16]. Respectively, insurance physicians working for the SSA conduct the work disability assessments for three groups of individuals falling under different work disability regulations. First, insurance physicians assess the disability for employed sick-listed workers, which constitutes a single conversation after two years of sick-leave from work (Dutch Social Security Schemes: Work and Income (Capacity for Work) Act). Second, sick-listed individuals without an employer receive guidance and assessment by an insurance physician already earlier during the first two years of their sick leave (Sickness Benefits Act). And, third, young disabled persons, who became disabled or chronically ill before the age of 18, receive a single assessment on their work opportunities by an insurance physician to determine (partial) work ability and eligibility for a disability claim (Young Disabled Persons Act).

### 2.2 Participants

Using convenience sampling, participants were initially recruited through the network of the research team by personal invitation through e-mail (*n* = 6). Additionally, the involved students recruited participants through their personal network (*n* = 1). Individuals were eligible to participate in the study if they were working as a ME within the SSA, performing work disability assessments in any scheme for at least one year.

The included participants (*n* = 7) consisted of 6 female and 1 male, of which 6 were registered MEs and 1 ME was a resident in training. The number of years working in the position of ME for the SSA ranged from longer than 10 years (*n* = 4), between 5 and 10 years (*n* = 2), and less than 5 years (*n* = 1).

### 2.3 Data collection

Semi-structured individual interviews (*n* = 7) lasting approximately one hour were conducted in May and June 2022, through a video call platform (either Zoom or Google Meet). All interviews were conducted by students under supervision of the research team (MM, NZ). The students conducted the interviews in pairs, alternating the role of the primary interviewer and note taker. The interviews were performed in either English (*n* = 6), or Dutch (*n* = 1), depending on the native language of the primary interviewer and preference of the interviewee. All interviews were audio recorded with the permission of the participants and were transcribed verbatim. An interview guide was used listing open-ended questions for general guidance during the interviews. The full interview guide can be found in Appendix A.

### 2.4 Data analysis

Thematic coding was performed for all individual interviews in three steps [17]. First, for each transcript open codes were assigned to all relevant text fragments by the first and second author (MH, NZ). Second, relations between the codes and larger concepts were identified by the second author (ZT), subdivided into barriers, facilitators and opportunities, and checked by the first and last author (MH, NZ). Facilitators were defined as factors that were mentioned currently adding value for the client during the work disability assessment, barriers were defined as factors that were mentioned as currently obstructing value for the client during the work disability assessment and opportunities were defined as factors that were mentioned as potentially adding value for the client during the work disability assessment in the future. Third, the identified themes were inductively subdivided into main themes in a phase of interpretation and explanatory construct by discussion (MH, NZ). The last two steps were conducted by using the online platform Miro (www.miro.com), an online whiteboard for visual collaboration. For all steps disagreements were resolved by discussion.

### 2.5 Role of the researchers and ethical considerations

Most of the involved students had conducted interviews prior to this study. However, they were not familiar with the process of a work disability assessment. Therefore, the students (incl. ZT) were supported by senior researchers (MM, NZ) to shape the aim and relevance of the study, and received guidance in the development of the interview guide. Authors MH, MM, JH, SB and NZ are experienced researchers within the field of occupational health and human-centred design and helped to further shape the aim and relevance of the study. Written informed consent was obtained from all participants by email. Ethical approval was obtained from the Medical Ethics Committee of the Amsterdam University Medical Center (number: W22_312 # 22.373).

## 3 Results

A large variety of facilitators (*n* = 22), barriers (*n* = 17) and opportunities (*n* = 11) to add value for the client during the work disability assessment from the perspective of the ME were identified, inductively subdivided into four main themes classified to add value during the work disability assessment: 1) coherent process, 2) interdisciplinary collaboration, 3) client-centred interaction and 4) information provision on the work disability assessment process. Below, we present the identified facilitators, barriers and opportunities for each of the four main themes. An overview of the identified facilitators, barriers and opportunities for each of the main themes, including representative quotes, is presented in Table 1.

**Table 1:** Representative quote for each of the identified facilitators, barriers and opportunities to add value clients during the work disability assessment from the perspective of the medical examiner (ME)

Theme	Subtheme	Quote
**1) Coherent process:** Includes all time related aspects to ensure a valuable work disability assessment process.		
**Facilitators**	Flexibility in consultation form	“[The ME] gets the opportunity to choose what is the best way to do this consultation, whether it’s face-to-face or on the phone.” –pt 3
	Use of communication skills	“If you have a lot of time [during the consultation], but you’re not asking the right things and not using like motivational or [other] techniques or something like that, then it is very difficult to help [clients].” —pt 3
	Involvement of team support	“[The social medical nurse] prepares the consultation. So, [they] look at the medical information. (..) So that when the ME starts the consultation, the necessary information is already available.” - pt 7
	Involvement of Case managers	“[The case manager] says what to do with [a file]. And he expects me to react. So that the process [of the client] continues faster.” —pt 2
**Barriers**	Laws and regulations	“It’s still difficult because we have a lot of rules and laws, so it’s not that I can help clients always how they want to be helped” —pt 3
	Bureaucratic character of the SSA	“We work for the [SSA], which is related to the government. So, it’s a governmental institution and that makes it very administrative” —pt 2
	Lack of medical information	“What I want as an insurance physician: you want all information about the reason of being sick listed, the medical history, but also related to work. But often this information is lacking.” —pt 4
	Information exchange by written letters	“Well, sometimes I speak to [the medical specialists] by phone, but mostly on paper. And this causes a delay [in the information exchange].” —pt 4
	Insufficient IT support	“Also a big problem in insurance medicine is that the [IT systems] are not working properly.” —pt 4
	Shortage of MEs	“I think like 25% of the assessment we can’t do because of a deficit of MEs” —pt 2
**Opportunities**	Shared-decision making	“And then, we can do our jobs, just like the occupational physicians, [meet with] clients regularly and then make a plan together with the clients on how to return to work.” —pt 6
	Refining the administrative requirements	“A report needs to be very extensive. But that is because of rules that have been imposed, and there are rules that are imposed by law. You can’t do anything about these unless the law is changed. But, there are also rules that we have imposed by ourselves. There might be some time savings by reporting or recording in a different way, so that it takes just a little less time and the process can go a little more efficiently.” —pt 7
	Acquiring all medical information prior to the consultation	“So sometimes if I have information beforehand, it’s not necessary to even do a consultation or like, pick up the phone and make some small phone calls to explain or to ask something. So (..) you can work more efficiently.” —pt 3
	Task delegation to other experts	“So, in another way you could also look at whether a labour expert or another employee could already conclude something from the contents of a client’s file before [the ME] looks into it from a medical point of view, if another route can be taken.” —pt 7
**2) Interdisciplinary collaboration:** Includes all aspects related to collaboration between the ME and other healthcare professionals to ensure a valuable work disability assessment process.		
**Facilitators**	Opportunities for collaboration with other disciplines	“Then I have to ask the clients and, my consultation will be, a bit longer and it will be more work for me with some. (..) Well, [the case manager] sends a letter to the occupational health physician or another physician to, get this information.” —pt 4
	Current collaboration with the labour expert form the SSA	“It is a bit different with the Sickness act, of course you have reintegration options there, and as a doctor, you can have an opinion about those reintegration options whether it is used properly and whether it is appropriate in the situation. And you do that together with the labour expert, because he also plays an important role in that reintegration.” —pt 7
	Discuss cases with colleagues	“If I have doubts or I do not know exactly how I will address this problem, I can consult with my colleague and then I learn from my colleague and the colleague learns from me.” —pt 1
**Barriers**	Strict division between medical roles	“The MEs are not curative, at the end of the 19th century they were excluded [IM] from the curative care. So that means that you are not involved in medical treatments anymore.” —pt 1
	Privacy regulations	“But the problem is that [information exchange] is difficult because of [the clients] privacy. If you want medical information, it is very hard to get it from other physicians” —pt 3
	SSA teams are too large	“What I see is now that the [SSA] teams are very big and everybody’s like swimming around and nobody knows from each other what they’re doing.” —pt 3
	Lack of understanding of each other’s roles and interests	“We sometimes don’t understand each other’s language, because I’m working with [functional ability] and [the clinicians] work with complaints and diseases. And sometimes, they don’t understand what we’re asking, because they don’t know the legislations and the consequences of that.” —pt 2
	Lack of knowledge were to find and how to contact others	“From a lot of [other professionals] I do not get one point of contact. So that’s very difficult. Especially when you’re not working at the same working place. (..) Who do you have to call.” —pt 3
**Opportunities**	Lower the threshold to find other stakeholders	“So if we would work in another way where we would have [..] frequent meetings with all of the disciplines involved, like for example once a week, every week on Monday, I think it would be better. And it would enhance the collaboration” –pt 6
	Improving communication with employers	“Maybe if [the employer] understood [the client’s situation] better, then, she would’ve kept her job” —pt 1
3) **Client-centred interaction:** Includes all aspects related to the supportive interplay from the ME towards the client to ensure a valuable work disability assessment process.		
**Facilitators**	Sufficient time during the consultation	“[The ME] has a lot of time for people, because you can talk for an hour and you can deepen all the problems very well.” —pt 3
	Guide the clients in its acceptance process	“It’s more guidance in accepting [the situation]. [To help the client to] be honest about the situation.” —pt 1
	Trustful relationship	“What is important to me in this is that [the client] feels heard, and that you [as the professional] also take [the clients situation] seriously.” —pt 7
	Focuses on finding meaning in the clients’ life	“So I think, for everybody it’s good to work and it’s not good to have a sickness benefits, actually. (..) [Clients] have to get purpose [in life]” —pt 3
	Motivational approach	“Because [the client] was like: No, I don’t want anything. I was like: But you have to try it. And I know, I was motivating him. So he said: Okay, I’ll do it for you.” —pt 2
	Holistic view on the personal situation	“You’re looking at the person as a whole. So not just the disease, but also what are the effects on [the client] mentally? What are the effects for the household and the partner, of course. So it’s the bigger picture.” —pt 6
	Offer interventions	“And then, I must take steps to ensure that she will go into another circuit. To try to get her into training or reeducation to get another type of job.” —pt 1
	Impartial assessment	“For me personally, the most important thing is that I feel, that I have captured the client’s functional capabilities as objectively as possible. And do as much justice as possible to their situation.” —pt 7
	Minimizing the inter-doctor variation	“We try as much as possible to keep that inter-doctor variation as small as possible for everyone, anywhere in the Netherlands.” —pt 7
	Offer opportunity to contact the ME after consultation	“And sometimes I will tell them that usually people that are very insecure during the consultation, or like with memory problems, they can, after the consultation, contact me to give additional information.” –pt 5
	Second opinion on the outcome of the assessment	“[Clients] have the right to object to the outcome of the assessments.” —pt 6
**Barriers**	Clients act hesitant and suspicious	“They don’t like [the ME] a lot at the beginning.” —pt 2
	Limited moments of contact	“They see me just once in a lifetime.” —pt 2
	Value of society	“I would like to share. The moral and ethical complication is that you do not work for the client. Your task is for the society. We have a societal task to better apply the laws, doing justice.” –pt 1
	Late starting point of contact	“It is well known that in the first three months after being sick listed, you can do the most regarding return to work. And now, I’m often seeing people after six months or even after two years.” —pt 4
	Clients lack the motivation and willingness to RTW	“And of course, there’s also clients’ responsibility, because they could have been more proactive. But, there’s not that much control of their behavior and if they are looking for work.” —pt 5
**Opportunities**	Earlier moments of contact	“Well, then, as insurance physicians, we can also have contact with clients in the first year of sick leave and not just, at the time of the assessments.” —pt 6
	More frequent moments of contact	“Ideally, in my opinion, we would be more like general practitioners where we can tell someone: Okay, we’ll see you next month again.” —pt 5
	More available manpower	“I think, (..) the client is not guided very well. So I think it’s better if there is somebody or more people who can do that job to really guide him.” —pt 3
	Financial security during RTW	“I think, it’s better if they get like a sickness benefit and with the opportunity to work. But only if it doesn’t work to get back on the sickness benefit.” —pt 3
**4) Information provision on the work disability assessment process**: Includes all aspects regarding information provision during the work disability assessment process towards the clients to ensure a valuable work disability assessment process.		
**Facilitators**	Clarify future functional capacities	“I translate my idea of how I think [the client] can [participate in] work into functional capacities.” —pt 2
	Clarify the assessment process	“So the most important thing is to take the clients by their hand and explain everything that you do during the assessment and what possible outcomes can be.” —pt 6
**Barriers**	Complicated structures in the laws and regulations	“For people with high education the whole process with all the legislations, is already very, very, difficult and complex.” —pt 2
**Opportunities**	Inform clients about the full process at the start of the entire service	“So, [clients] are a little bit afraid or they have a lot of stress about it [the insecurity of the process]. They don’t know how it works and nobody’s going to contact them. So I think, they will be better if they will get informed in the beginning.” —pt 3

MEs = medical examiners; RTW = return to work; SSA = social security agency.

1) **Coherent process**: Includes all time related aspects to ensure a valuable work disability assessment process.
  ***Facilitators:*** The MEs indicated to be ‘*flexible in how they carry out the consultation*’, face-to-face or by phone, enabling them to better meet the client’s personal preferences. Besides, the MEs highlighted the importance to ‘*use communication skills*’ during the consultation to offer clients the opportunity to express themselves. In addition, ‘*involving team support and case managers*’ enhanced the efficiency of the process, benefiting the lead time for the clients. It was mentioned that team support and case managers were additional professionals that could support the ME during the work disability assessment.
  ***Barriers:*** ‘*Strict laws and regulations*’ were mentioned as a barrier for efficiency and coherency in the work disability assessment process at an individual level, since the MEs reported that the laws and regulations did not always meet the client’s individual needs. As MEs need to work according to these laws and regulations, they mentioned that generic laws do not always suit the personal situation of each client. Besides, the ‘*bureaucratic character of the SSA*’ was mentioned to lead to a lack of flexibility and ability to take individual needs into account when assessing work disability. Additionally, bureaucracy was reported to add to the administrative burden of the MEs. The MEs also indicated that the efficiency during the consultation was hindered due to a ‘*lack of medical information*’ about the client. Medical information was not always available at the time of the consultation which may limit the coherency in the work disability assessment process. Currently, the request for information exchange from the ME, and information provision by the curative care professionals is done through ‘*written letters*’ by postal mail, which was reported to reduce the efficiency of the process significantly. In addition, optimal information exchange between the professionals within the SSA was reported to be limited due to ‘*insufficient IT support*’ offering limited digital solutions being a barrier for an efficient information flow. Furthermore, increased waiting times were mentioned due to a ‘*shortage of MEs*’.
  ***Opportunities:*** One ME suggested that they can better meet the client’s needs if they could provide more continuous and coherent support with ‘*shared-decision making*’ together with the client in terms of the return to work (RTW) plan of a client. Thereby, multiple MEs suggested that they could save time by reducing and ‘*refining the administrative requirements*’ within the SSA in the way the MEs are obligated to report their work disability assessment under the prevailing social insurance legislation, but also reviewing current working methods as imposed by the professional organization of Dutch insurance doctors. ‘*Acquiring all medical information of the client prior to the consultation*’ would support better efficiency of the process as having the full picture of the medical situation could benefit the quality of the consultation. Furthermore, MEs suggested to make the process more coherent by the introduction of ‘*task delegation to other experts*’ within the work disability assessment process. The MEs stated that allocating tasks such as gathering medical information to occupational health nurses could lead to efficiency gains for MEs during consultation with clients.
2) **Interdisciplinary collaboration:** Includes all aspects related to collaboration between the ME and other healthcare professionals to ensure a valuable work disability assessment process.
  ***Facilitators:*** The MEs mentioned that existing ‘*opportunities for collaboration with other disciplines*’ could enhance the reliability of their work disability assessment. Through collaboration the information flow may be enhanced, and the quality of the assessment could be better tailored to the personal situation and needs of clients. ‘*Current collaboration with the labour expert from the SSA*’ was mentioned to smooth the process for the assessment. Besides, MEs highlighted the importance of ‘*discussing cases with colleagues*’ to deliberate on difficult cases and in turn influence the quality of their assessment.
  ***Barriers:*** Due to the assessing nature in the task of the ME, in the Netherlands there is a ‘*strict division between the medical roles*’ of curative and occupational healthcare professionals. Therefore, MEs indicated that ‘*privacy regulations*’ obstruct their ability and possibility to collaborate with the curative care sector. MEs reported that information exchange is not possible without written approval by the client due to the privacy regulations restricting information flow between social security and curative healthcare. Another barrier mentioned by the MEs was that the ‘*SSA teams working together were experienced as too large*’ in terms of the size of the team, causing inefficient collaborations within the teams. Creating smaller teams may have a positive influence on the efficiency and accessibility for collaboration. Collaboration with professionals outside the SSA, as professionals from curative healthcare, was reported to be limited because the MEs mentioned a ‘*lack of knowledge about the role and interests*’ in the work disability assessment process by these professionals. Since the social security is separated from the curative care, it was mentioned that it was not always clear to the MEs what the interests of other stakeholders may be. Additionally, a ‘*lack of knowledge on where to find and how to contact other stakeholders*’ was reported as limiting collaboration in a practical manner.
  ***Opportunities:*** In order to be able to improve value for clients through more efficient collaboration between different professionals, MEs indicated that it was important to ‘*lower the threshold to find other stakeholders*’, for example by providing contact details in advance or scheduling fixed moments for reciprocal contact. Moreover, besides improving the communication with curative care professionals, MEs also mentioned the added-value of ‘*improving communication with employers*’ at an earlier stage of sick leave of the clients in order to facilitate better understanding at the side of the employer, which can facilitate flexibility and willingness at the employers’ side to facilitate earlier RTW for the client or accommodation of alternative working positions.
3) **Client-centred interaction:** Includes all aspects related to the supportive interplay from the ME towards the client to ensure a valuable work disability assessment process.
  ***Facilitators:*** Even though most clients only visit the ME once, this consultation for assessing their functional limitations and work capacity was reported to last one hour on average. MEs indicated that the duration of an hour offers them ‘*sufficient time during the consultation*’ to listen to the client and to develop a ‘*trustful relationship*’ with the client. In addition, during this consultation the MEs mentioned the importance to ‘*guide the clients in its acceptance process*’ and ‘*focus on finding meaning in the client*’*s life*’, for example by applying a ‘*motivational approach*’ to activate the client’s awareness in their own RTW process. Hereby, MEs indicated their ‘*holistic view on the personal situation*’ as valuable for the client. Besides, MEs indicated that one of the most powerful factors to create value for the clients was the opportunity to ‘*offer interventions*’ as, for example, additional physiotherapy or reintegration programs to facilitate RTW. MEs reported conducting an ‘*impartial assessment*’ of the client’s functional abilities, as it is pre-defined in professional guidelines associated with the law, being able to do justice to the individual situation of the client. However, one participant referred to this impartial assessment as a barrier for client-centred interaction since following guidelines does not always allow for accounting for individual needs in the outcome of the assessment. Besides, it was mentioned that in the work disability assessment process there was a focus on ‘*minimizing the inter-doctor variation*’ to maintain the quality, and add value, as the MEs need to comply to strict rules for the assessment. If clients felt insecure about their capacities to RTW, one ME indicated that a facilitator for more client-centred interaction would be to ‘*offer the opportunity to contact the ME after consultation*’ if they had any more questions in order to let them feel more assured. Furthermore, MEs mentioned that a higher level of self-directed care was offered for clients by the possibility for a ‘*second opinion on the outcome of the assessment*’. This could give clients the possibility to speak-up and receive a more suitable assessment if they think the outcome did not fit their personal situation.
  ***Barriers:*** Because of the importance of the assessment for clients due to possible financial impact, MEs reported that ‘*clients start to act hesitant and suspicious*’ towards the ME, limiting the abilities to build-up a trustful relationship with the clients. This, in turn, could hinder the ability to provide a client-centred assessment as MEs might not receive all needed information from the client. Feeling mutual trust is a prerequisite for being open during the consultation. This was mentioned to be even more enhanced by the fact that a large part of the clients have ‘*limited moments of contact*’ with the ME, often only once. However, since the ME is not only responsible for the value for the individual clients, but also protects the ‘*value for society*’ with fair distributions of public funds for disability benefits, the MEs mentioned that they cannot always meet the needs of the individual client with the societal impact in mind. Especially for clients working for an employer at the start of their sick leave, meeting the ME only after a two-year period of sick leave, the MEs highlighted a ‘*late starting point of contact*’ as a barrier to add value through reintegration guidance since the ‘*clients lack motivation and willingness to RTW*’ after these two years and mutual trust could not be developed. In this case it was mentioned that it was hard for MEs to let the client realize the added value to RTW. After a two-year period a single consultation hour may not lead to the desired impact to motivate clients to RTW.
  ***Opportunities:*** MEs suggested that value could be created by shifting the strict focus on assessing the functional abilities towards more additional guidance to RTW, which could be supported by ‘*earlier moments of contact*’ with the clients, ‘*more frequent moments of contact*’ and ‘*more available manpower*’ of professionals. Besides, ME indicated the expectation that extra ‘*financial security during RTW*’ would decrease uncertainty for clients, and thereby may enhance their willingness and motivation to RTW as well as influence the trust in the professional.
4) **Information provision on the work disability assessment process:** Includes all aspects regarding information provision during the work disability assessment process towards the clients to ensure a valuable work disability assessment process.
  ***Facilitators:*** Multiple participants highlighted that MEs offered good information provision to the clients by thoroughly ‘*clarifying the assessment process*’ to the client during the consultation. It was mentioned that by explaining what the client can expect regarding follow-up appointments and ‘*clarify expectations regarding the client*’*s future functional capabilities*’ MEs could add value for the client.
  ***Barriers:*** It was mentioned by MEs that additional clear information provision was needed since ‘*complicated structures in the existing work disability laws and regulations*’ make it hard for clients to understand the legislations and to know what to expect within the process, which may cause stress.
  ***Opportunities:*** One ME suggested that an opportunity to reduce stress levels for the client would be to make sure that clients were ‘*informed about the full process already at the start of the entire service*’ before they had their first consultation with an ME. It was mentioned that transparent information may be beneficial to reducing stress for the clients and therefore contribute to adding value for clients.

**Fig. 1:**
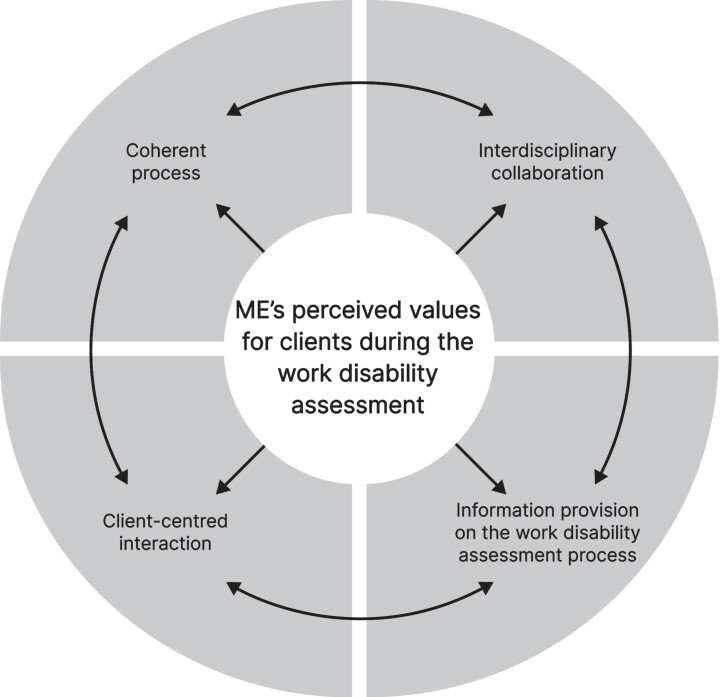
Representation that the four main themes indicated as valuable for the client within insurance medicine by the professionals are all interrelated with each other. MEs = medical examiners.

The four main themes presented above are deemed to be closely related, as illustrated in Fig. 1. It is suggested that, for example, interdisciplinary collaboration can result in a more coherent process, better client-centred interaction and a more complete information provision on the work disability assessment process. While the other way around, for example, a more complete information provision on the work disability assessment process results in a more coherent process, better interdisciplinary collaboration and supports better client-centred interaction. Thus, it is important to not see the presented main themes as separate entities when interpreting the results and trying to add value in practice.

## 4 Discussion

This study identified four main themes on how MEs add value for clients during the work disability assessment; 1) coherent process, 2) interdisciplinary collaboration, 3) client-centred interaction, and 4) information provision on the work disability assessment process. For each of these main themes factors adding value for the client as well as barriers for adding value as perceived from the perspective of the MEs were explored, including opportunities to overcome the barriers.

### 4.1 Agreements and disagreements with other studies

The four main themes identified in this study are in line with a previous qualitative systematic review identifying clients’ values within occupational healthcare from the clients’ perspective (Hagendijk ME, et al. unpublished data), suggesting that the MEs interviewed in this study had a good understanding of what clients consider important during the work disability assessment process. An earlier systematic review also showed that, besides the expected benefits of adding value for clients [3–5], curative care professionals also benefited from more professional engagement, joy in practice and job satisfaction [18]. On the other hand, aspects important to professionals such as concerns regarding available time and challenges in team work may occur, being a barrier to add value [18].

While in this study MEs stated the need for collaboration with other professionals outside the SSA, literature confirms this need for more in-depth discussion with the ME from the occupational physicians’ perspective to contribute to a more efficient process for clients [19]. However, earlier attempts to improve the clients’ RTW process were not successful due to poor existing collaboration and differences in interest between the SSA, vocational rehabilitation agencies and healthcare providers [20]. Additionally, better information exchange between MEs and occupational physicians was not found to significantly influence RTW for clients [21]. Based on this study, it is suggested that better information exchange between those two professional groups may be of added-value for the efficiency in the process, but does not add value for clients in terms of faster RTW [21]. In addition, previous literature confirmed a lack of inclusivity in society for individuals needing an adapted working position, stating that subsidized jobs are rare [22], which supports the suggested opportunity in this study to create more value for clients by encouraging the societal system to be more inclusive.

To add value for clients by client-centred interaction during the work disability assessment, in previous studies MEs indicated that consultations should last longer and should be planned more frequently to establish a good relationship [22]. However, in agreement with the findings in this study, the MEs indicated to not have the means to offer this extra support because of a limitation imposed under the current Dutch laws and regulations [22] and due to a shortage in MEs as found in this study. In addition, in this study it was indicated that clients may have initial negative feelings towards MEs as a barrier for client-centred interaction. In previous studies, this was suggested to be caused by wrongful expectations of the social security system by the clients [22]. However, the MEs indicated that showing understanding and respect and creating a trustful relationship with the client is valuable during the work disability assessment. In previous studies, MEs highlighted that entering the social security system in general has a certain tone to assess a client creating a more distant and impersonal approach [22]. Also when studying the clients’ experiences, clients highlight the negative feeling that the ME does not act in their interest, but in the interest of society [23]. Moreover, while the MEs in this study plead that their broad knowledge and holistic view adds value for their clients, the value-based healthcare concept which describes how to add value within curative care advocates for specialization in a certain client group [24], suggesting that the way of adding value within occupational health and curative care can deviate from each other.

Recent literature confirms the finding that complicated structures in the laws and regulations make it hard for clients to understand the process [23]. Also, in coherence with the findings from this study, it was found that clients experience the information provision regarding the work disability assessment process as negative [23]. Consequently, in both literature and our study, it was suggested that clients’ experiences with receiving information on the work disability assessment process can be improved by better information provision on the process at the start of the service [23]. Therefore, it was suggested that future improvement on better information provision can lead to higher value for clients.

In agreement with the barriers to add value for clients during the work disability assessment identified in this study, professionals in curative care also identified barriers for the delivery of valuable curative care including unjustified client expectations, lack of professional knowledge and skills, a lack of collaboration between professionals and infrastructure issues [25]. Earlier literature studying the application of evidence-based medicine during the work disability assessment, which focuses on improving client-centred care by explicit and judicious use of current best evidence in making decisions about the care of individual clients, found that a lack of time, lack of skills of the professional and the existing legislation are existing barriers [26].

### 4.2 Methodological considerations

A principal limitation in this study was the small sample size, increasing the possibility that full saturation was not reached in the identified themes. However, according to the high number of subthemes, we believe that despite this low sample size the most important themes to add value for clients during the work disability assessment were identified. Possible inter-interviewer variance might have influenced the reliability, caused by each student being the primary interviewer only once. However, the impact of this was kept limited through a general interview guide used throughout all interviews. Conducting the interviews via an online video call platform may have contributed positively to the variety in participant characteristics, allowing inclusion of participants with a larger geographical distribution and might have thus limited selection bias. No negative selection bias by online interviewing was expected, since it was assumed that all MEs are experienced in conducting video calls due to experience with video-calling during the Covid19 pandemic. Moreover, the extensive thematic analysis executed by the experienced researchers was considered a methodological strength.

### 4.3 Implications for future research

In this study we only included MEs working for the SSA, responsible for allocating disability benefits on behalf of the government assessing employees, unemployed and young disabled. The generalizability of our findings towards the private sector allocating disability benefits for self-employed workers may be limited due to differences in the occupational healthcare system and access to work disability insurance for these clients. In addition, while the values of employees within occupational health has been extensively researched [23], the perspective of clients on their own values is underrepresented for self-employed clients. Therefore, further research should investigate these factors to add value as well as barriers for work disability assessments in the private sector from both a professional and client perspective.

Although, this study identified the factors adding value as well as barriers to add value for clients during the work disability assessment from the perspective of the ME, it may be interesting to study the generalizability of these identified factors and barriers to add value for other professionals involved in the clients’ occupational healthcare process to facilitate the provision of valuable care over the full cycle of occupational healthcare including other professional groups as well. Besides, to facilitate provision of real client-centred occupational healthcare, further research should focus on the clients’ perspectives on the identified factors adding value during a work disability assessment, and to what extent these values are met in current occupational healthcare. Insights may provide information on the most important factors and barriers to add value and thereby improve the clients’ value in current occupational healthcare.

### 4.4 Implications for practice

Although this research took place in the specific context of work disability assessments in The Netherlands, a context which contains a unique division in medical roles between occupational and curative care professionals, it is assumed that most findings are transferable to the context of occupational healthcare in general. In addition, the focus on adding value for clients is in line with the current shift towards a more value driven healthcare provision [7], making the findings of this study important for policy makers on how to apply better value driven care during the work disability assessment and occupational healthcare. The suggested opportunities already highlight potential solutions for some of the factors identified as barriers to add value. Furthermore, the overview of the factors stimulating and obstructing a value-driven work disability assessment might help MEs to improve value for their clients in their practice, stimulating overall better value-driven occupational healthcare provision.

## 5 Conclusion

The identified possible facilitators, barriers and opportunities to add value during the work disability assessment for the client from a ME’s perspective provides insight in what MEs consider as valuable in their work, what they consider as barriers to add value for their clients, and what they think are possible opportunities to increase the value for the clients. This overview may stimulate to remove inefficiencies in the practice of the work disability assessments, as well as it may stimulate improvements in the current work disability assessment practice, in order to better match the clients’ needs and, thereby, add value for the client.

## Ethical approval

Ethical approval was obtained from the Medical Ethics Committee of the Amsterdam University Medical Center (number: W22_312 # 22.373).

## Informed consent

Written informed consent was obtained via e-mail from all participants.

## Conflict of interest

The authors declare that they have no conflict of interest.

## Appendix A - interview guide

**Part 1: General questions / Getting to know each other (± 6 min)** Examples of questions:

– Could you please tell me who you are, where you work, and how many years of experience you have as an insurance physician?
– What made you become an insurance physician?
– How would you describe your job in a few sentences?
– What do you consider important in your job? And what is less important?

**Part 2: Discussing collaborations (± 15 min)** Examples of questions:

– How would you describe the relationship with that [name another professional]?
– What makes this collaboration/relationship valuable to you?
– What are the advantages and disadvantages to work with the disciplines of your team? And what are the advantages and disadvantages of your position?
– If you could, what would you change about your collaborations in the future? Why?

**Part 3: An example of a client’s journey (± 10 min)** Examples of questions:

– Can you tell us a story of success for you; in which you may have had a difficult time at the beginning with the client, but that ended up in a good way?
– What was your goal to achieved with this client?
– What is most valuable/ most important for you in this journey?
– What do you perceive to be valuable for a client?

**Part 4: Some last questions (± 4 min)** Examples of questions:

– Does it differ per patient-group / case what is the most valuable outcome? Could you give some examples?
– Is there anything else that you would like to share with us about your work as an Insurance Physician and what you find valuable?
